# Special Issue: Application of Photoactive Nanomaterials in Degradation of Pollutants

**DOI:** 10.3390/ma12152459

**Published:** 2019-08-02

**Authors:** Roberto Comparelli

**Affiliations:** CNR-IPCF, Istituto Per i Processi Chimici e Fisici, S.S. Bari, c/o Dip. Chimica Via Orabona 4, 70126 Bari, Italy; roberto.comparelli@cnr.it

**Keywords:** photocatalysis, nanomaterials, advanced oxidation processes, water treatments, recalcitrant pollutants, gas-phase pollutants, NO_x_, VOCs, building materials, disinfection

## Abstract

Photoactive nanomaterials are receiving increasing attention due to their potential application to light-driven degradation of water and gas-phase pollutants. However, to exploit the strong potential of photoactive materials and access their properties require a fine tuning of their size/shape dependent chemical-physical properties and on the ability to integrate them in photo-reactors or to deposit them on large surfaces. Therefore, the synthetic approach, as well as post-synthesis manipulation could strongly affect the final photocatalytic properties of nanomaterials. The potential application of photoactive nanomaterials in the environmental field includes the abatement of organic pollutant in water, water disinfection, and abatement of gas-phase pollutants in outdoor and indoor applications.

## 1. Introduction

In recent years, one of the most important concerns of the scientific community and society has been health and environmental protection via the smart and sustainable use of natural resources. In this context, water resources are gaining increasing attention due to the occurrence of emerging pollutants including dyes, pharmaceutical and personal care products, endocrine disruptors, and pathogens [1,2]. Moreover, the increasing amount of atmospheric pollutants has been regarded among the main causes of respiratory diseases such as emphysema and bronchitis, which arise from the contact of NOx and lungs [3]. Unfortunately, conventional pollution remediation methods show limited performances. For instance, in the field of water treatment adsorption or coagulation, such methods aim to concentrate pollutants by transferring them to other phases for example, sedimentation, filtration, chemical, and membrane technologies involve high operating costs and can generate toxic secondary pollutants in the ecosystem [4] and chlorination, although widely used in disinfection processes, can generate by-products associated with cancer or other pathologies [5].

It turns out that the interest of the scientific community has been focusing on alternative methods such as the “advanced oxidation processes (AOPs)”. AOPs are convenient and innovative alternatives to conventional wastewater and air treatment processes aiming to accomplish the complete mineralization of organic pollutants (i.e., their conversion into safe by-products such as O_2_, H_2_O, N_2_, and mineral acids) [6,7].

Among AOPs, semiconductor-based photocatalysis has recently emerged as a promising air/water treatment [8]. Photocatalysis takes place upon the activation of a semiconductor with electromagnetic radiation from sun or artificial light. When exposed to electromagnetic radiation, a semiconductor absorbs photons with sufficient energy to inject electrons from the valence band (VB) to its conduction band (CB), generating electron hole pairs (e^−^/h^+^). The h^+^ have an electrochemical potential sufficiently positive to generate ^•^OH·radicals from water or to directly oxidize many organic pollutants adsorbed onto the semiconductor surface, while the e^−^ react with oxygen molecules to form superoxide anions, ^•^O_2_^−^, that quickly react with H^+^ to finally produce ^•^OH radicals after a series of concatenated reactions in water or can directly reduce target molecules adsorbed on the surface [9]. 

Nanostructured catalysts have demonstrated improved performances with respect to its bulk counterpart, thanks to their extremely high surface-to-volume ratio that turns into a high density of catalytically active surface sites. In addition, due to the size-dependent band gap of nanosized semiconductors, it is possible to fine tune the redox potentials of photogenerated electron–hole pairs to selectively control photochemical reactions. Furthermore, charges photogenerated in nanocatalysts can easily reach the catalyst surface, thus decreasing the probability of bulk recombination [10]. Nonetheless, a large-scale application of nanosized photocatalysts for environmental purpose is still hampered by technological issues and by high costs related to its capability to obtain photoactive nanocatalysts with a high reaction yield and adequate morphological and structural control [11]. 

The aim of the present special issue is to report on recent progress towards the application of photoactive nanomaterials and nanomaterials-based coatings in pollutants degradation, paying particular attention to cases of study close to real application: Scalable synthetic approaches to nanocatalysts, preparation of nanocatalyst-based coatings, degradation of real pollutants and bacteria inactivation, and application in building materials.

## 2. This Special Issue

This special issue consists of one review and six research articles. The review from Petronella et al. reports a selection of synthetic approaches suitable for a large-scale production of mesoporous TiO_2_-based photocatalysts. Attention has been focused on mesoporous TiO_2_ due to its unique features, which include a high specific surface area, improved ultraviolet (UV) radiation absorption, high density of surface hydroxyl groups, and a significant ability for further surface functionalization. The overviewed synthetic strategies have been selected and classified according to the following criteria: (i) High reaction yield, (ii) reliable synthesis scale-up, and (iii) adequate control over morphological, structural, and textural features. The potential environmental applications of such nanostructures include water remediation and air purification which are also discussed [12].

The research article from Professor Mascolo and co-workers demonstrates the effectiveness of the novel multiphasic hydroxyapatite–TiO_2_ material (HApTi) for the photocatalytic treatment of diclofenac. Diclofenac is one of the most detected pharmaceuticals in environmental water matrices and it is recalcitrant to conventional wastewater treatment plants. The authors investigated the toxicity of transformation products by using different assays: Daphnia magna acute toxicity test, Toxi-Chromo Test, and the *Lactuca sativa* and *Solanum lycopersicum* germination inhibition test. Overall, the toxicity of the samples obtained from the photocatalytic experiment with HApTi decreased at the end of the treatment, showing the potential applicability of the catalyst for the removal of diclofenac and the detoxification of water matrices [13]. 

Professor Yurdakal’s group demonstrated the synthesis of Pt-loaded TiO_2_ nanotube on Ti anode by anodic oxidation in ethylene glycol. Such an approach allowed the control of the length of the nanotube as a function of anodic oxidation time. The obtained materials were exploited in photoelectrocatalytic, electrocatalytic, and photocataytic degradation of Paraquat, one of the most widely used herbicides. The obtained results evidenced that the photoanodes show a significant synergy for photoelectrocatalytic activity [14].

A simple and low-cost method to preparing hybrid photocatalysts of copper (I) oxide/titania is proposed in the paper by Professor Kowalska and co-workers. They investigated the photocatalytic and antimicrobial properties of prepared nanocomposites in three reaction systems: Ultraviolet-visible (UV-Vis) induced methanol dehydrogenation and oxidation of acetic acid, and 2-propanol oxidation under visible light irradiation. Furthermore, bactericidal and fungicidal properties of Cu_2_O/TiO_2_ materials were analyzed under UV, visible, and solar irradiation, as well as for dark conditions [15]. 

Cu_x_O thin films deposited using HiPIMS (high-power impulse magnetron sputtering) on polyester under different sputtering energies were successfully synthesized by Professor Rtimi and co-workers. The photocatalytic performance of the photocatalyst was evaluated for the degradation of a toxic textile dye (Reactive Green 12; RG12) under visible light LEDs irradiation. The recycling of the catalyst showed a high stability of the catalyst up to 21 RG12 discoloration cycles. ICP-MS showed stable ions’ release after the 5th cycle for both ions. This allows potential industrial applications of the reported HiPIMS coatings in future [16].

Rehman et al. investigated the effects of TiO_2_ nanoparticles on the sulfate attack resistance of ordinary Portland cement (OPC) and slag-blended mortars. The results show that the addition of nano-TiO_2_ accelerated expansion, variation in mass, loss of surface microhardness, and widened cracks in OPC and slag-blended mortars. Nano-TiO_2_ containing slag-blended mortars were more resistant to sulfate attack than nano-TiO_2_ containing OPC mortars [17].

The research article from Liang et al. describes the synthesis and characterization of ZnO-ZnS core-shell nanorods by combining the hydrothermal method and vacuum sputtering. The results of comparative degradation efficiency toward methylene blue showed that the ZnO-ZnS nanorods with the shell thickness of approximately 17 nm had the highest photocatalytic performance. The highly stable catalytic efficiency and superior photocatalytic performance supports their potential for environmental applications [18].
